# Data on production and characterization of melamine-furan-formaldehyde particles and reversible reactions thereof

**DOI:** 10.1016/j.dib.2019.104056

**Published:** 2019-05-25

**Authors:** Katharina Urdl, Stephanie Weiss, Günter Hesser, Andreas Kandelbauer, Edith Zikulnig-Rusch, Uwe Müller, Wolfgang Kern

**Affiliations:** a Wood K Plus – Competence Center for Wood Composites & Wood Chemistry, Altenberger Straße 69, A-4040, Linz, Austria; b School of Applied Chemistry, Reutlingen University, D-72762, Reutlingen, Germany; c ZONA, Johannes Kepler University, Altenberger Straße 69, A-4040, Linz, Austria; d Chair in Chemistry of Polymeric Materials, Montanuniversitaet Leoben, A-8700, Leoben, Austria; e Polymer Competence Center Leoben GmbH, A-8700, Leoben, Austria

**Keywords:** Melamine-formaldehyde, Furan-functionalized particle, Diels-alder reaction, Thermoset

## Abstract

The data present in this article affords insides in the characterization of a newly described bi-functional furan-melamine monomer, which is used for the production of monodisperse, furan-functionalized melamine-formaldehyde particles, as described in https://doi.org/10.1016/j.eurpolymj.2019.04.006 Urdl et al., 2019. In the related research article Urdl et al., 2019 data interpretations can be found. The furan-functionalization of particles is necessary to perform reversible Diels-Alder reactions with maleimide (BMI) crosslinker to form thermoreversible network systems. To understand the reaction conditions of Diels-Alder (DA) reaction with a Fu-Mel monomer and a maleimide crosslinker, model DA reaction were performed and evaluated using dynamic FT-IR measurements. During retro Diels-Alder (rDA) reactions of the monomer system, it was found out that some side reaction occurred at elevated temperatures. The data of evaluating the side reaction is described in one part of this manuscript. Additional high resolution SEM images of Fu-Mel particles are shown and thermoreversible particle networks with BMI2 are shown. The data of different Fu-Mel particle networks with maleimide crosslinker are presented. Therefore, the used maleimide crosslinker with different spacer lengths were synthesized and the resulting networks were analyzed by ATR-FT-IR, SEM and DSC.

**Table:** Specifications table

Subject area	*Polymer Chemistry*
More specific subject area	*Furan-melamine particles and Diels Alder (DA) reactions with maleimides*
Type of data	*Images, graphs, figures and tables*
How data was acquired	***ATR-FT-IR****spectra were acquired with a Bruker Tensor 27 instrument. The IR spectra were processed using the software Origin 2017 64 bit and were unit vector normalised before analysis.* ***NMR****spectra were acquired by using a Bruker Avance II 400 instrument and resonance frequency of 400.*13 MHz *for*^*1*^*H. The data was analyzed using MestReNova.* *The****DSC****measurements were performed with a Mettler Toledo 822e instrument and the STAR® software was used for the evaluation of normalised enthalpies of the DSC traces. The data was replotted using the Origin 2017 64 bit software.* ***FIB-SEM****images were acquired by using a Fieldemission-SEM of ZEISS, Dualbeam 1540XB. All the samples were sputtered with 5 –* 10 nm *of gold.* ***SEM****images were recorded using a Phenom Pro X scanning electron microscopy, using an acceleration voltage of* 15 kV*.*
Data format	*Raw and analyzed*
Experimental factors	*Fu-Mel monomer, Fu-Mel particles and Fu-Mel particle-BMI networks are vacuum dried before analysis. Different BMI crosslinker were synthesized using spacer molecules from 148 to* 900 g mol^−1^.
Experimental features	*Fu-Mel monomer synthesis and particle production is described in*https://doi.org/10.1016/j.eurpolymj.2019.04.006[1]. *Fu-Mel monomer characterization using IR spectroscopy and*^*1*^*H-NMR spectroscopy are presented. DA reaction of the monomer reaction with BMI1 is evaluated in DMF and IR spectra data of the DA reaction are presented. The side reaction of melamine NH*_*2*_*and BMI compound is evaluated in DMF and the reaction is followed by IR spectroscopy. The data of DSC measurements on the side reaction are presented. Data on high resolution SEM images of the Fu-Mel particles and Fu-Mel particle networks are presented. Retro DA reaction of the dried network of Fu-Mel particles with BMI2 crosslinker were followed by IR spectroscopy. Data of DA networks with different kind of BMI crosslinker and Fu-Mel particles are analyzed using IR, SEM and DSC.*
Data source location	*Kompetenzzentrum Holz (Wood K Plus), Sankt Veit an der Glan, Austria*
Data accessibility	*data are available within this article*
Related research article	https://doi.org/10.1016/j.eurpolymj.2019.04.006[1]

**Table:** 

**Value of the data** • The data is valuable to other researchers as it shows additional information and characterization to the experimental results about reversible binding of Fu-Mel particles and maleimide crosslinker described in the research article https://doi.org/10.1016/j.eurpolymj.2019.04.006 [1]. • Other researchers in the community of self-healing materials and polymer materials, which are able to perform reversible binding, can benefit from this data as it shows the characterization of thermoreversible Diels-Alder chemistry. • The present data helps other researchers to better understand the reaction conditions for a successful Diels-Alder reaction. It further helps the reader of reference [1] to understand and detect the ongoing side reaction of maleimides at elevated temperatures and to avoid it. • The data shows, how the side reaction of maleimides can be followed by FT-IR and DSC measurements, which should help other researchers to detect the side reaction

## 1 Data

The shown data is supplementary the article of https://doi.org/10.1016/j.eurpolymj.2019.04.006 [1].

**Fig. 1:**
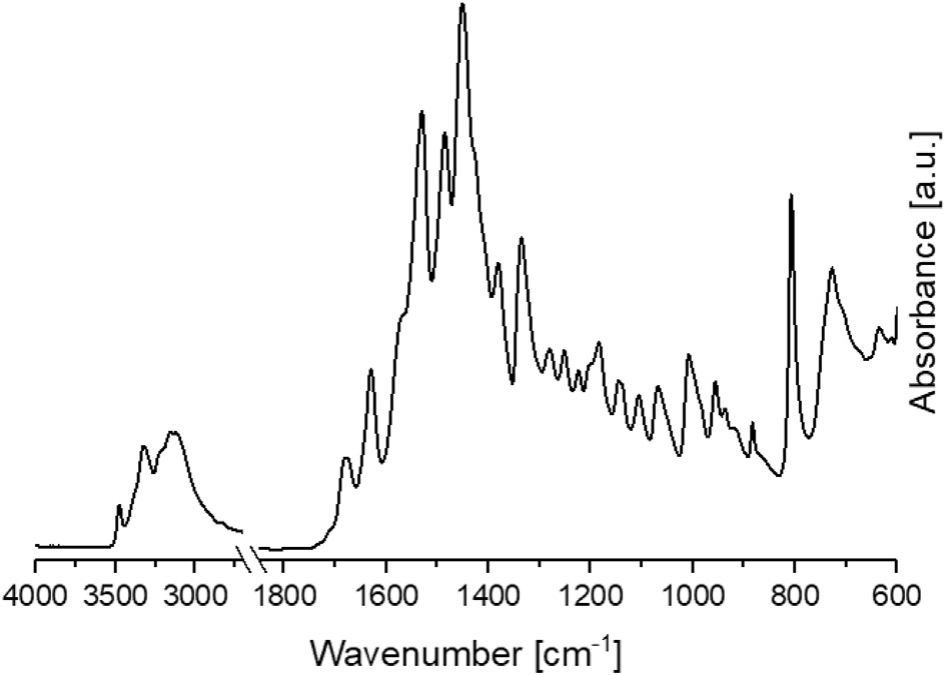
IR spectra of Fu-Mel; Spectra were recorded in a range of 4000–600 cm^−1^ with a spatial resolution of 4 cm^−1^. The spectra was plotted with Origin software.

**Fig. 2:**
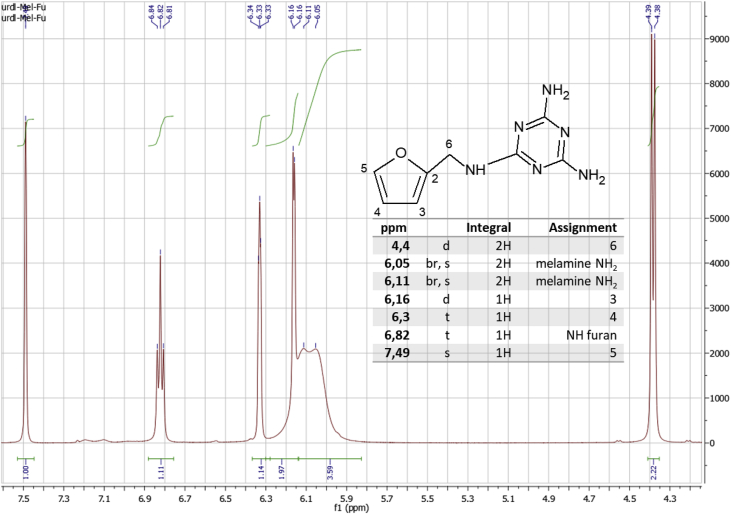
^1^H NMR in d_6_ - DMSO of Fu-Mel, the chemical structure and the peak assignment. Spectra were acquired by using a resonance frequency of 400.13 MHz, with standard Bruker pulse programmes. The spectra were evaluated using MestReNova software. Chemical shifts were given in ppm, referenced to residual solvent signals (DMSO: 2.49 ppm for ^1^H).

The data in Fig. 1, the ATR-FT-IR spectrum and in Fig. 2, the ^1^H NMR of the bi-functional monomer Fu-Mel is shown, which confirms the chemical structure presented also in Fig. 2.

**Fig. 3:**
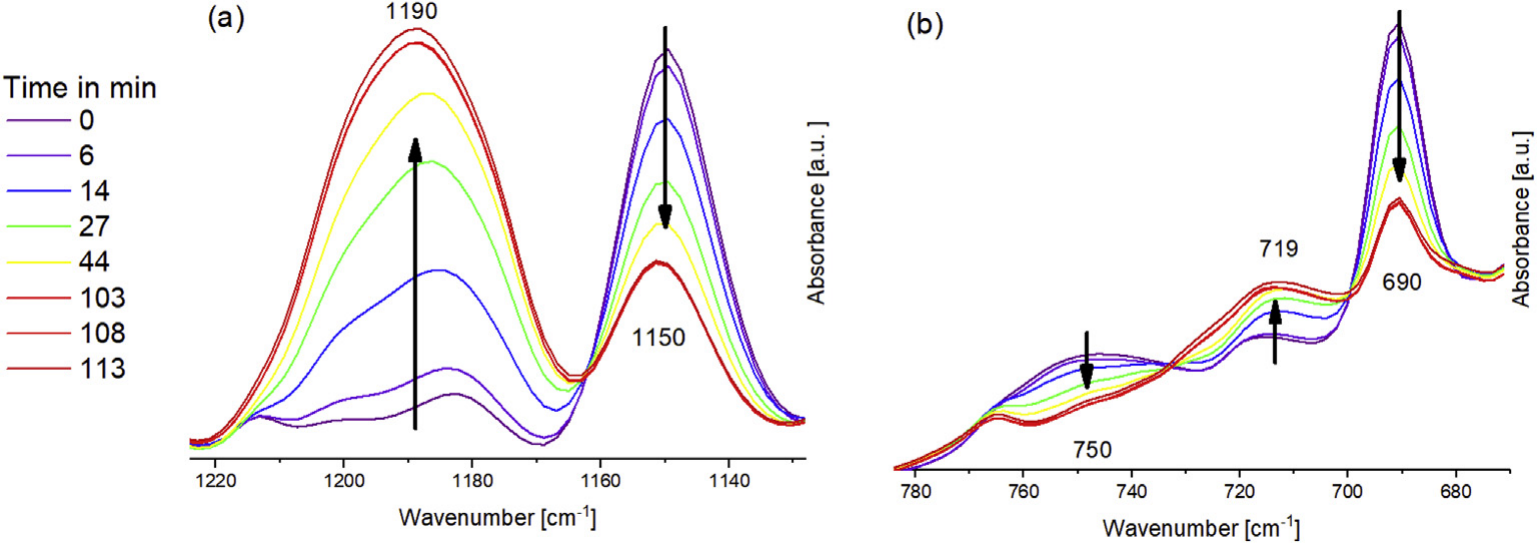
IR spectra during DA reaction between Fu-Mel and BMI1 in DMF at 60 °C; Right: 1220–1120 cm^−1^; Left: 850–670 cm^−1^. Spectra were recorded in a range of 4000–600 cm^−1^ with a spatial resolution of 4 cm^−1^. The spectra were unit vector normalised and plotted with Origin software.

**Fig. 4:**
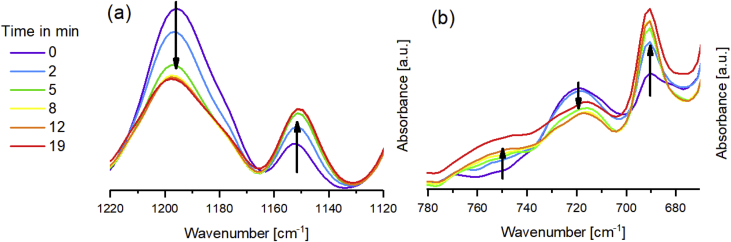
IR spectra during rDA reaction between Fu-Mel and BMI1 in DMF at 105 °C; Right: 1220–1120 cm^−1^; Left: 780–660 cm^−1^. Spectra were recorded in a range of 4000–600 cm^−1^ with a spatial resolution of 4 cm^−1^. The spectra were unit vector normalised and plotted with Origin software.

**Fig. 5:**
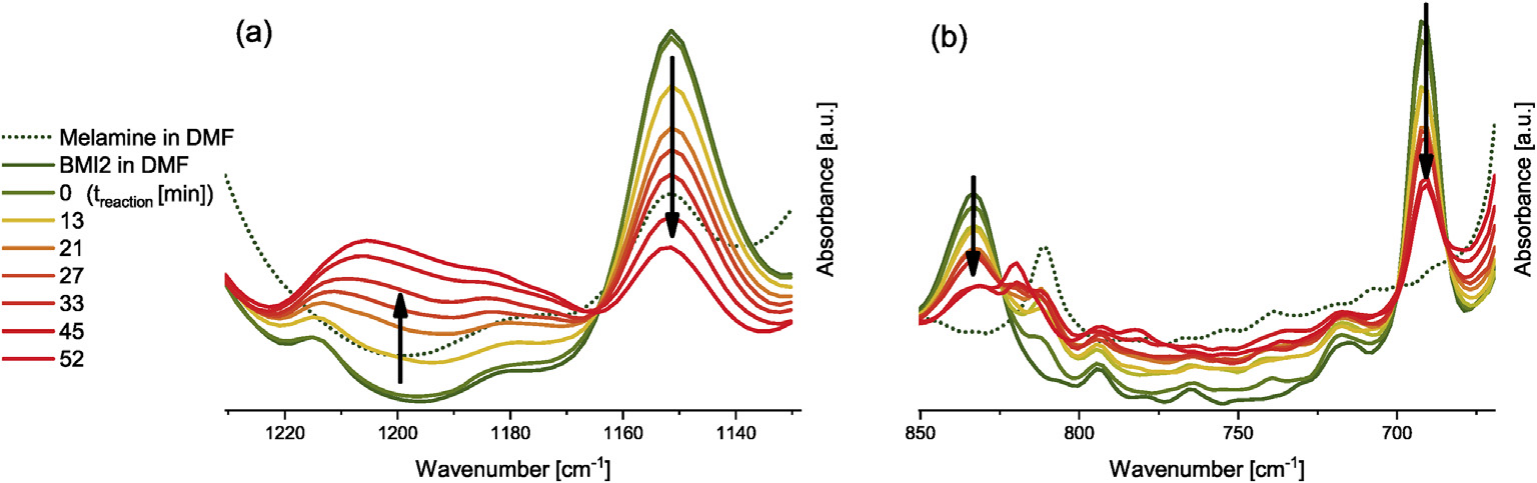
IR spectra of pure melamine in DMF (green dotted) and pure BMI in DMF (green line). IR spectra while heating melamine and BMI2 in DMF isothermally at 120 °C after 0, 13, 21, 27, 33 45 and 52 minutes of reaction time. Spectra were recorded in a range of 4000–600 cm^−1^ with a spatial resolution of 4 cm^−1^. The spectra were unit vector normalised and plotted with Origin software.

**Fig. 6:**
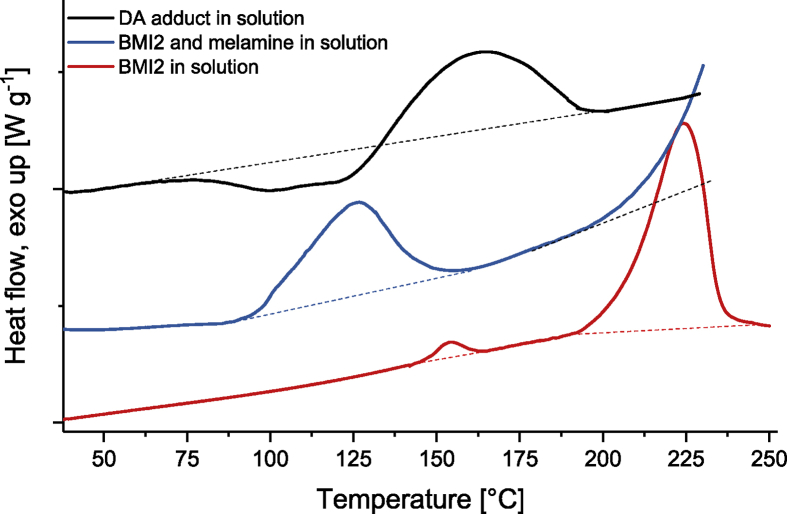
DSC of BMI2 and melamine in solution (blue) and BMI2 in solution (red). The samples were measured in high pressure steel (gold-plated) DSC pans, with a heating program from 25 to 250 °C at a scanning rate of 3 °C min^−1^ under nitrogen atmosphere. The normalised enthalpy integrals of the exothermal signals ΔH were integrated using the STAR 8.10 software package. The traces were replotted using the Origin software.

The relevant peak variations to follow the Diels-Alder (DA) reaction of the Fu-Mel monomer with a maleimide crosslinker (BMI1) in DMF is shown in Fig. 3. The data in Fig. 4 shows the retro Diels-Alder (rDA) reaction. During rDA reaction at temperatures of 120 °C (as described in [1]) a side reaction of the BMI was detected. It was found out, that NH_2_ groups on the Fu-Mel react with the BMI C=C bond to initiate a dimerisation of the BMI, which consumes the DA reactive maleimide ring structure. The ATR-FT-IR data, while following the reaction of pure melamine and BMI2 in DMF, are shown in Fig. 5. Data obtained by DSC measurements of the reaction of melamine with BMI2 solution and only BMI2 in solution are presented in Fig. 6. The data in Table 1 shows the exothermal experimentally measured reaction enthalpies for the exothermal events of melamine and BMI in solution and only BMI in solution. Theoretical reaction enthalpies for the proposed side reaction, as described in [2], are also presented in Table 1.

**Table 1:** Experimental determined reaction enthalpies for the exothermal events of DSC traces in Fig. 6 and the theoretical calculated due to the reaction mechanism in [2].

Sample	Exothermal event			ΔH_exp._ [kJ/mol]	ΔH_theor._ [kJ/mol]
	T_start_ [°C]	T_end_ [°C]	Heat flow [J/g]		
DA network in solution [1]	137	180	−160	−165	
BMI2 and melamine in solution	105	145	−236	−167	−175
BMI2 in solution	190	250	−400	−233	

**Fig. 7:**
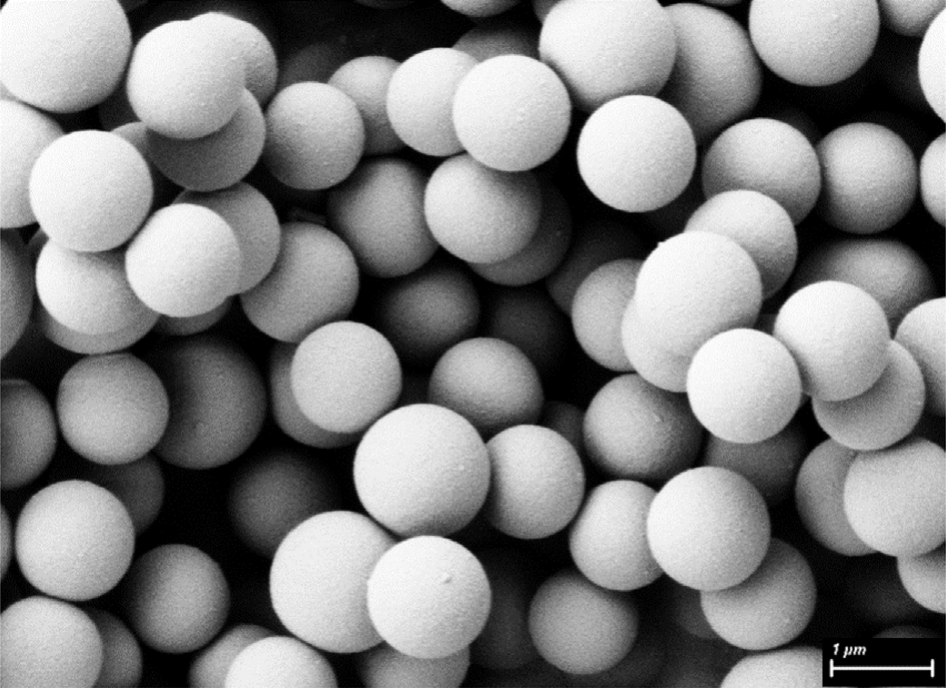
FIB-SEM Image of Fu-Mel particles. The particles were sputter coated with 5–10 nm gold, and images were recorded using a charge reduction sample holder with an acceleration voltage of 3 kV.

High-resolution FIB-SEM images of Fu-Mel particles are presented in Fig. 7.

**Fig. 8:**
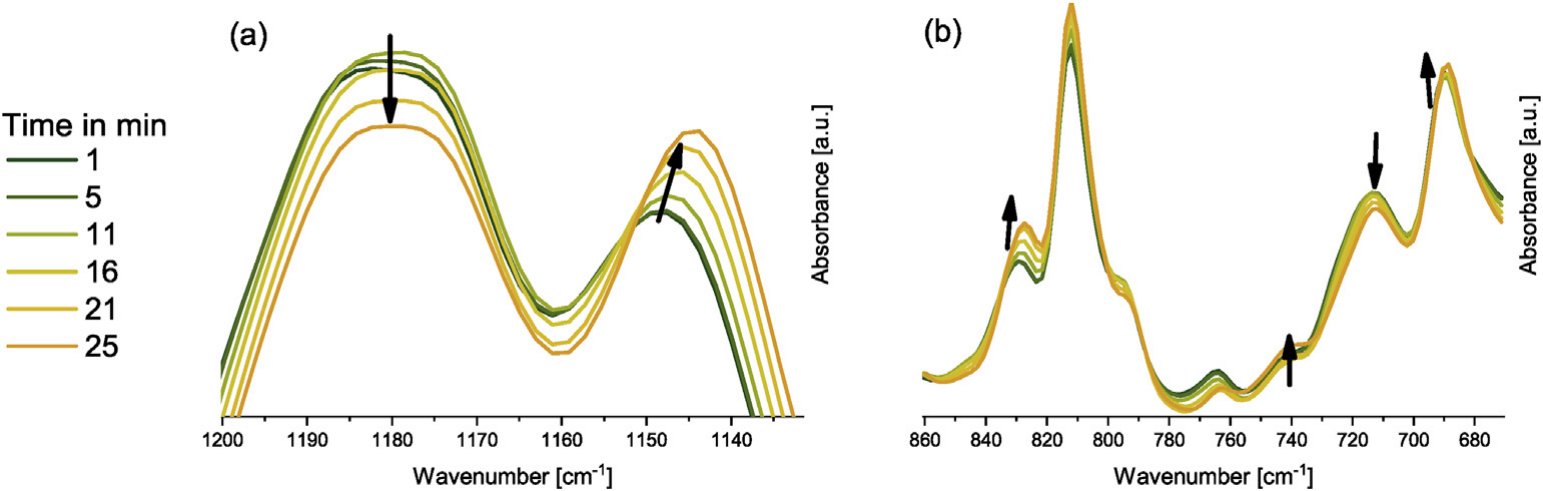
IR spectra during rDA reaction of the solid DA network of Fu-Mel particles with BMI2 crosslinker at 120 °C; Right: 1220–1120 cm^−1^; Left: 840–660 cm^−1^. Spectra were recorded in a range of 4000–600 cm^−1^ with a spatial resolution of 4 cm^−1^. The spectra were unit vector normalised and plotted with Origin software.

The data presented in Fig. 8 show the relevant peak areas of the rDA reaction of the DA network of Fu-Mel particles with BMI2 crosslinker.

**Fig. 9:**
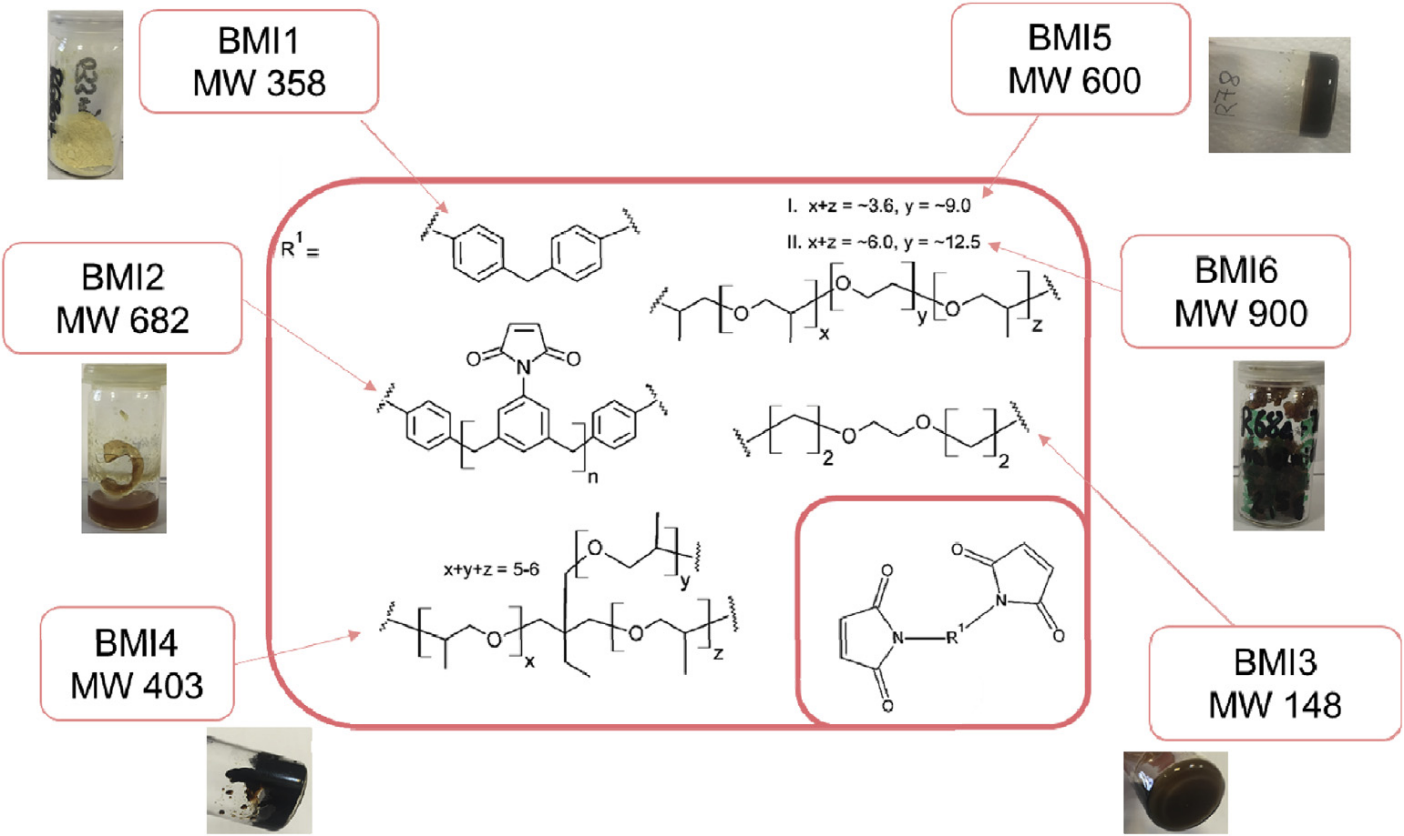
Chemical structure (molecular weights of spacer = MW) and pictures of Fu-Mel particle BMI networks with different spacer lengths BMI crosslinker.

**Fig. 10:**
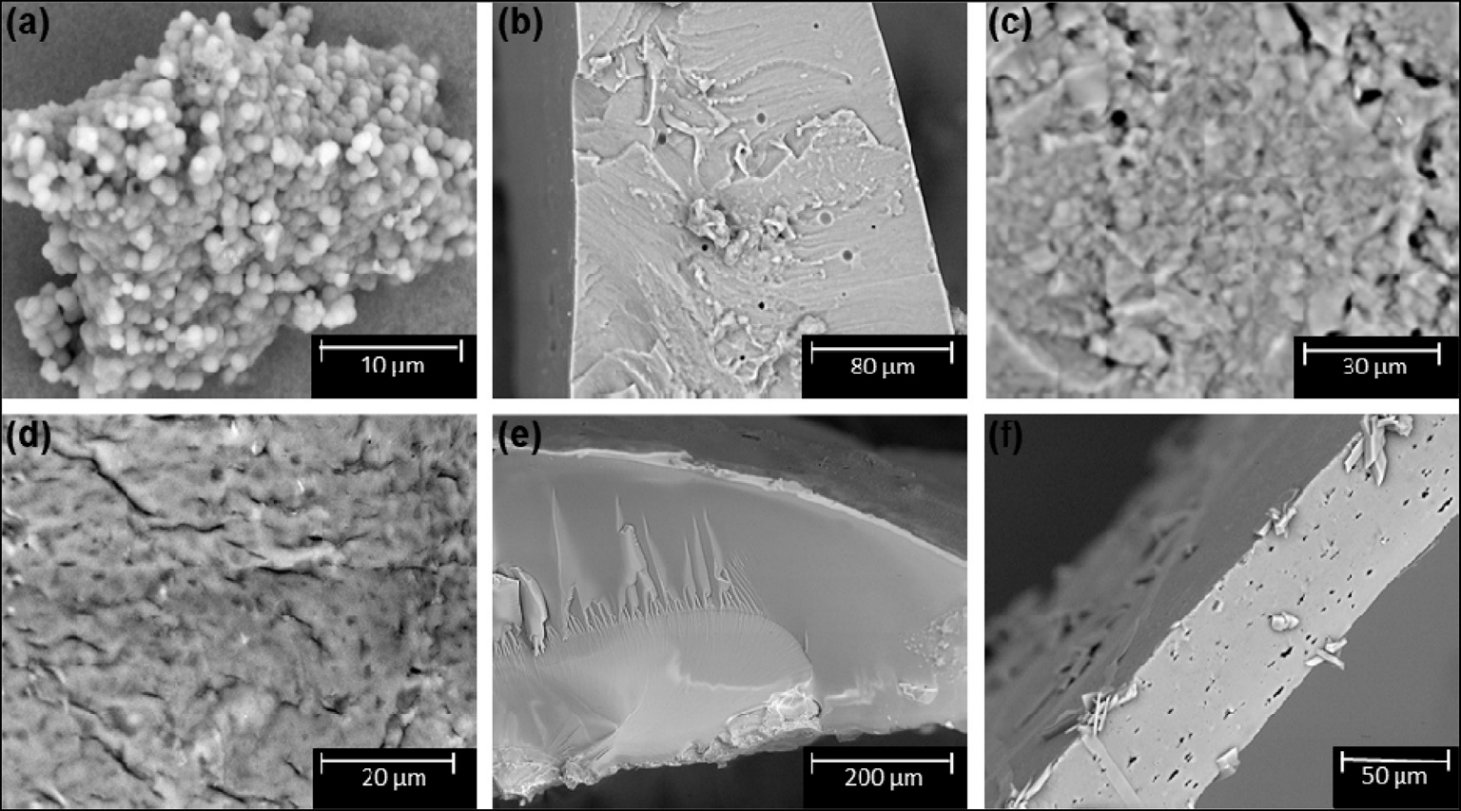
SEM images of the vacuum dried DA networks between Fu-Mel particles and (a)BMI1; (b) BMI5; (c) BMI6; (d) BMI4; (e) BMI2; (f) BMI3. SEM images were recorded using a charge reduction sample holder with an acceleration voltage of 15 kV.

**Fig. 11:**
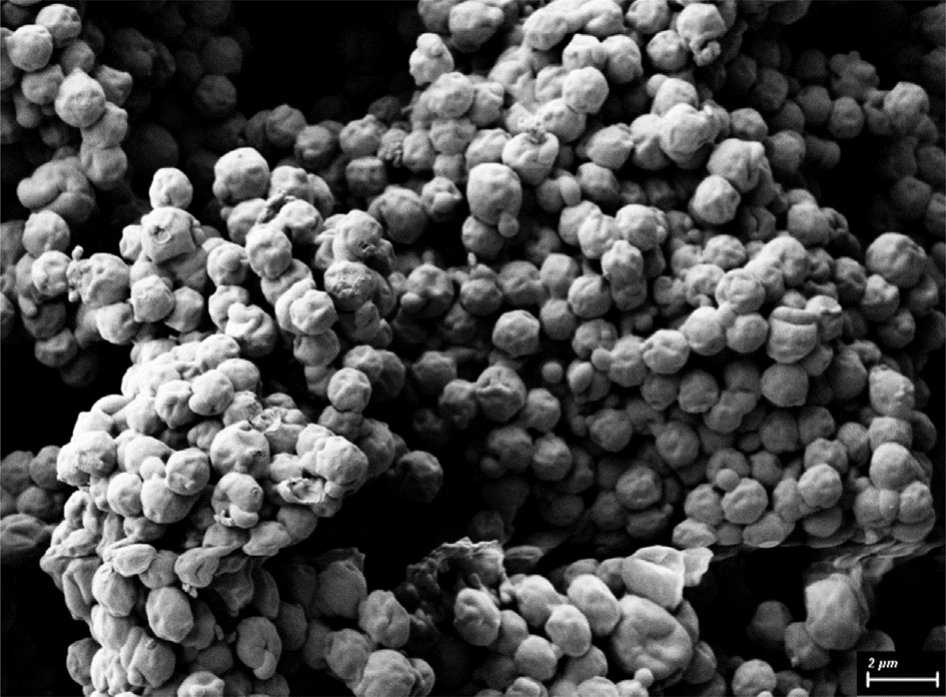
FIB-SEM Image of DA networks of Fu-Mel particles with BMI1. The particle networks were sputter coated with 5–10 nm gold, and images were recorded using a charge reduction sample holder with an acceleration voltage of 3 kV.

**Fig. 12:**
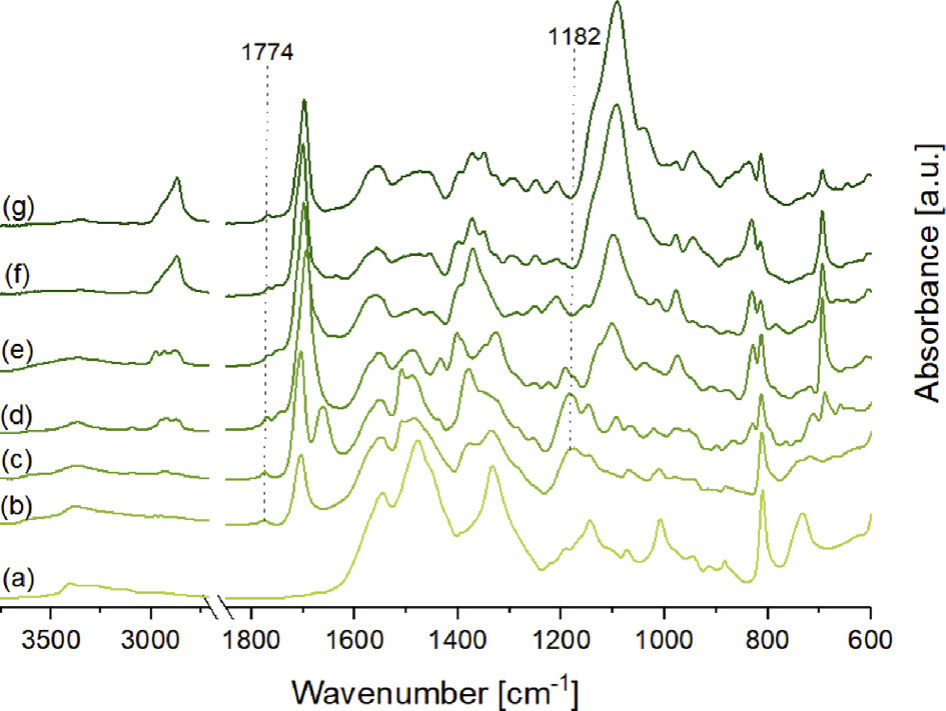
IR spectra of (a) Fu-Mel particles; (b–g) IR spectra of the vacuum dried DA networks between Fu-Mel particles and different BMI crosslinker (b) BMI1; (c) BMI2; (d) BMI3; (e) BMI4; (f) BMI5; (g) BMI6. Spectra were recorded in a range of 4000–600 cm^−1^ with a spatial resolution of 4 cm^−1^. The spectra were unit vector normalised and plotted with Origin software.

**Fig. 13:**
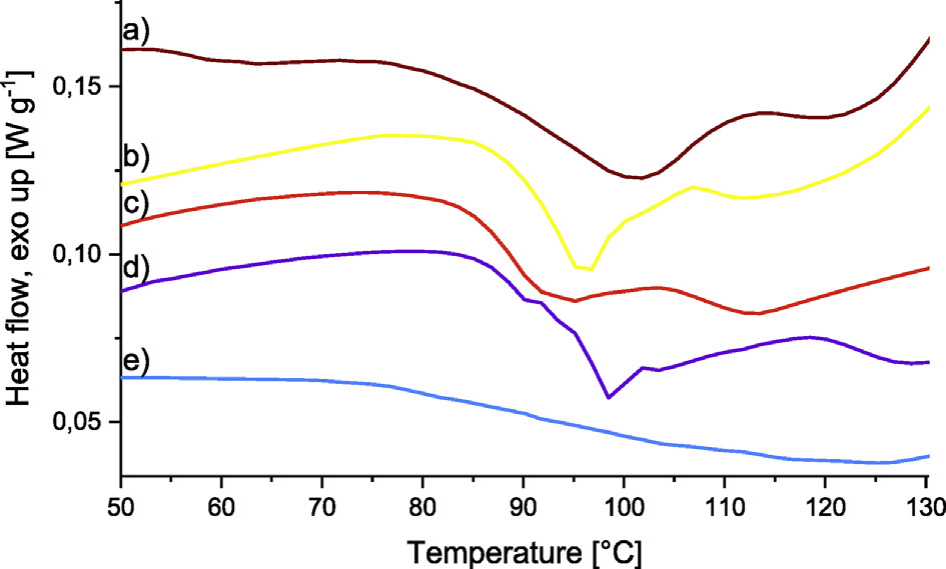
DSC traces of vacuum dried DA networks between Fu-Mel particles and different BMI crosslinker (a) BMI1; (b) BMI2; (c) BMI4; (d) BMI6; (e) BMI5. The samples were measured in high pressure steel (gold-plated) DSC pans, with a heating program from 25 to 130 °C at a scanning rate of 3 °C min^−1^ under nitrogen atmosphere. The traces were replotted using the Origin software.

Pictures of the networks of Fu-Mel particles with BMI crosslinker are presented in Fig. 9, where also the molecular structure of the used BMI crosslinker is shown. The molecular weight and the used spacer chemicals of the different termed BMI crosslinkers are described in Table 2. The SEM data presented in Fig. 10 show the microstructure of the DA networks of Fu-Mel particles with BMI crosslinker of Fig. 9. High-resolution FIB-SEM images of Fu-Mel particle-BMI1 networks are presented in Fig. 11. ATR-FT-IR spectra of the DA networks are presented in Fig. 12 and the DSC curves are shown in Fig. 13.

**Table 2:** Sample names of the used maleimide crosslinker with different spacer lengths.

Name	Spacer Chemical	Molecular weight (MW) [g mol^−1^]
BMI1	Diphenylmethane (HOS Technik)	358
BMI2	Polyphenylmethane (HOS Technik)	682
BMI3	Jeffamine^®^ EDR-148	148
BMI4	Jeffamine^®^ T-403	403
BMI5	Jeffamine^®^ ED-600	600
BMI6	Jeffamine^®^ ED-900	900

## 2 Experimental design, materials and methods

### 2.1 Materials

All experimental design, materials and methods were based on reported paper [1].

Sodium bicarbonate, ethanol (70%), maleic acid anhydride, chloroform, trimethylamine, acetic anhydride and acetone for the maleimide crosslinker synthesis, and N,N-dimethylformamide (DMF) as a solvent for DA reactions, were purchased from Carl Roth GmbH & Co KG (Karlsruhe, Germany). 4,4′-Diphenylmethane bis-maleimide (BMI1) and polyphenylmethane bis-maleimide (BMI2) were obtained from HOS-Technik GmbH (Austria). Jeffamine^®^ EDR-148, T-403, ED-600 and ED-900 were obtained from Huntsman Corporation. All chemicals were used as received.

### 2.2 Methods

*Synthesis of N*^*2*^*-(furan-2-ylmethyl)-1,3,5-triazine-2,4,6-triamine (Fu-Mel)*

The synthesis of the bi-functional monomer is already described in [1].

*Fu-Mel pre-polymer preparation and particle preparation*

The pre-polymer preparation and the particle production procedure is already described in [1].

*Preparation of maleimides (BMI) with different spacer lengths*

Maleimides (BMI) with different sizes of spacers were prepared by using different Jeffamines^®^ and reacting them with maleic anhydride following a method reported in the literature [3]. A solution of maleic anhydride (50 mmol) in 50 mL of chloroform was kept under nitrogen at 10 °C. The Jeffamine^®^ (25 mmol if EDR-148, ED-600, ED-900; 16.7 mmol if T-403) was added dropwise over a period of 3 h and thereafter the mixture was allowed to warm up to room temperature under stirring for 2 h. The solution was then dried under vacuum and the maleamic acid was obtained as a yellow viscous liquid. Then, the maleamic acid (4.6 mmol), triethylamine (3.0 mmol), and sodium acetate trihydrate (3.5 mmol) were stirred in 10 mL of acetone under nitrogen. Acetic anhydride (29 mmol) was added and the temperature was raised to 70 °C for 2.5 h. Finally, the acetone was rotatory evaporated and the viscous solution (BMI) was dried under vacuum maintaining the temperature below 60 °C.

*Diels-Alder coupling of Fu-Mel particles with maleimide crosslinker*

0.5 mmol of the BMI and 0.5 g of the Fu-Mel particles were added to 10 mL DMF. The mixture was stirred at 60 °C for 24 hours. The particles and the BMI crosslinker formed a gel-like network. The obtained network was dried under vacuum overnight. The used BMI crosslinkers and pictures of the obtained networks are shown in Fig. 9.

For the spectroscopic analysis, the obtained networks were dried under vacuum overnight to yield a coating film. For dynamic ATR-FT-IR measurements, the particle network with BMI2 was placed on a heatable ATR stage and heated to 120 °C with a heating rate of 5 °C min^−1^. Spectra were taken every 60 seconds (32 scans, resolution 4 cm^−1^ in a spectral range of 4000–600 cm^−1^, Fig. 8.

*Evaluating the side reaction of melamine and BMI*

For the evaluation of the side reaction of NH_2_ groups in the melamine compound and the maleimide C=C moieties, 214 mg melamine and 340 mg BMI2 were placed in 3 mL DMF. The reaction solution was directly weighted (10 mg) into a high pressure steel (gold-plated) DSC pan (method see *Differential Scanning Calorimetry*). The reaction of melamine and BMI at isotherm 120 °C was followed by ATR-FT-IR, samples were taken from the reaction solution during the reaction (Fig. 5).

*ATR-FTIR spectroscopy*

ATR-FTIR spectroscopy measurements were performed with a Bruker Tensor 27 instrument. FT-IR spectra were recorded in the range of 4000–600 cm^−1^ versus air as the background spectrum with a resolution of 4 cm^−1^ and a scan rate of 32 scans per spectrum. The Fu-Mel spectrum, Fig. 1 was recorded by placing the isolated, dried reaction product on the ATR stage. Reaction monitoring of the DA reaction at isothermally heating at 60 °C (Fig. 3) was made by taking samples in defined time intervals (method described in [1]). rDA reaction was followed while isothermally keeping the sample at 105 °C (Fig. 4) while taking samples. DA networks of Fu-Mel particles with different BMI crosslinker were vacuum dried before measuring each sample, Fig. 12.

^*1*^*H NMR spectroscopy*

NMR spectra were recorded on a Bruker Avance II 400 (resonance frequencies 400.13 MHz for ^1^H) equipped with a 5 mm observe broadband probe head (BBFO) with z–gradients at room temperature with standard Bruker pulse programmes. The sample was dissolved in 0.6 ml of DMSO-d_6_ (99.8% D, euriso-top). Chemical shifts were given in ppm, referenced to residual solvent signals (DMSO: 2.49 ppm for ^1^H). ^1^H NMR data were collected with 32k complex data points. D_2_O-exchange was done by addition of some drops of D_2_O to the dissolved sample followed by extensive shaking prior to measurement of ^1^H spectra.

*Differential Scanning Calorimetry (DSC)*

All DSC traces were recorded by using a differential scanning calorimeter 822e DSC of Mettler Toledo (Greifensee, Switzerland). For the evaluation of the side reaction of melamine with BMI2, the sample was directly weighted (10 mg) into a high pressure steel (gold-plated) DSC pan. After hermetically sealing the pan, the sample was heated from 25 to 250 °C at a scanning rate of 3 °C min^−1^ under nitrogen atmosphere, Fig. 6. The enthalpy changes were recorded and analyzed for the peak start and end temperature. The normalised enthalpy integrals of the exothermal signals ΔH were integrated using the STAR 8.10 software package (Mettler Toledo, Greifensee, Switzerland). Fu-Mel particle BMI networks with different spacer lengths were measured by weighting the vacuum dried DA networks into high pressure steel (gold-plated) DSC pans (sample volume 5–10 mg). After hermetically sealing the pan, each sample was heated from 25 to 180 °C at a scanning rate of 5 °C min^−1^ under nitrogen atmosphere, Fig. 13.

*High-resolution scanning electron microscopy*

Shape and surface morphology of the isolated particles were observed by scanning electron microscopy (Fieldemission-SEM, ZEISS Dualbeam 1540XB, Jena, Germany). A small amount of the sample (Fu-Mel particles, Fig. 7 and Fu-Mel particle networks with BMI1, Fig. 11) was attached to the aluminium mounts using carbon adhesive tabs and sputter coated with 5–10 nm gold. SEM images were recorded using a charge reduction sample holder with an acceleration voltage of 3 kV.

*Scanning electron microscopy*

Morphology of the vacuum dried DA networks of the Fu-Mel particles with BMI crosslinker, Fig. 10, were observed by scanning electron microscopy (Phenom Pro X instrument, from Phenom, Eindhoven, Netherlands). A small amount of the sample was attached to the aluminium mounts using carbon adhesive tabs. SEM images were recorded using a charge reduction sample holder with an acceleration voltage of 15 kV, Fig. 10.
